# Modulation of Growth and Mycotoxigenic Potential of Pineapple Fruitlet Core Rot Pathogens during In Vitro Interactions

**DOI:** 10.3390/toxins16080344

**Published:** 2024-08-07

**Authors:** Manon Vignassa, Christian Soria, Noël Durand, Charlie Poss, Jean-Christophe Meile, Marc Chillet, Sabine Schorr-Galindo

**Affiliations:** 1 CIRAD, UMR Qualisud, F-97410 Saint-Pierre, Réunion, Francechristian.soria@cirad.fr (C.S.); meile@cirad.fr (J.-C.M.); marc.chillet@cirad.fr (M.C.); 2 Qualisud, Univ Montpellier, Avignon Université, CIRAD, Institut Agro, IRD, Université de La Réunion, Montpellier, France; noel.durand@cirad.fr (N.D.); charlie.poss@cirad.fr (C.P.); 3 CIRAD, UMR Qualisud, F-34398 Montpellier, France

**Keywords:** *Ananas comosus*, beauvericin, co-culture, fruitlet core rot, fumonisins, *Fusarium*, in vitro, LC/MS-MS, *Talaromyces*

## Abstract

Pineapple Fruitlet Core Rot (FCR) is a fungal disease characterized by a multi-pathogen pathosystem. Recently, *Fusarium proliferatum*, *Fusarium oxysporum*, and *Talaromyces stollii* joined the set of FCR pathogens until then exclusively attributed to *Fusarium ananatum*. The particularity of FCR relies on the presence of healthy and diseased fruitlets within the same infructescence. The mycobiomes associated with these two types of tissues suggested that disease occurrence might be triggered by or linked to an ecological chemical communication-promoting pathogen(s) development within the fungal community. Interactions between the four recently identified pathogens were deciphered by in vitro pairwise co-culture bioassays. Both fungal growth and mycotoxin production patterns were monitored for 10 days. Results evidenced that *Talaromyces stollii* was the main fungal antagonist of *Fusarium* species, reducing by 22% the growth of *Fusarium proliferatum*. A collapse of beauvericin content was observed when FCR pathogens were cross-challenged while fumonisin concentrations were increased by up to 7-fold. Antagonism between *Fusarium* species and *Talaromyces stollii* was supported by the diffusion of a red pigmentation and droplets of red exudate at the mycelium surface. This study revealed that secondary metabolites could shape the fungal pathogenic community of a pineapple fruitlet and contribute to virulence promoting FCR establishment.

## 1. Introduction

Pineapple Fruitlet Core Rot (FCR) is a major issue in the export market, causing severe fruit quality depreciation [1]. The particularity of the FCR pathosystem is that healthy and diseased fruitlets coexist in the same pineapple fruit with no external symptoms. For several years, FCR was studied considering *Fusarium ananatum* as the main causal agent even though other species, *Fusarium proliferatum*, *Fusarium oxysporum*, and *Talaromyces stollii*, were identified in both healthy and infected fruitlets [2,3,4]. The pathogen(s) benefit from pineapple sugar accumulation and in some cases, successfully counter host defenses, leading to fruitlet flesh browning, a so-called ‘black spot’ [5]. Recently, *F. proliferatum*, *F. oxysporum*, and *T. stollii* were found to induce similar symptoms to those caused by the infection of *F. ananatum*, thereby confirming their implication in the FCR pathosystem [6]. The mycobiomes of healthy and diseased fruitlets contain the four FCR pathogens as the most abundant fungal species, suggesting that pathogenesis does not rely solely on fruitlet contamination. Additionally, in planta studies of FCR pathogens found significant changes in fungal community structure that are induced following inoculation with the pathogenic *Fusarium* spp. [6]. This requires a transition from an endophytic to a pathogenic behavior related to the increase in fungal biomass in the host tissues.

In plant diseases, strains of *F. proliferatum* and *F. oxysporum* species are widely studied for their ability to produce mycotoxins, which are harmful to human health [7,8,9,10,11]. The biohazard of these compounds led to the establishment of maximum levels in food and feed products by the European Food Safety Authority (EFSA) [12]. Considering FCR incidence across several pineapple production countries, isolates of *Fusarium* species obtained from FCR-diseased fruits were identified as producers of fumonisin B_1_ (FB_1_), fumonisin B_2_ (FB_2_), fumonisin B_3_ (FB_3_), moniliformin (MON), and beauvericin (BEA) under in vitro culture conditions [13]. Thereafter, FB_1_ and BEA were measured at higher concentrations in naturally diseased or *Fusarium*-inoculated fruitlets when compared to healthy fruitlets, suggesting that these toxins could be implicated in FCR fungal virulence [14]. In pineapple, pre- and post-harvest conditions are defined as influent parameters for both FCR pathogenesis and the introduction of mycotoxins in the food chains [6,15,16,17]. In fruit production and processing schemes, the main safety issues related to mycotoxins are patulin, ochratoxin A, aflatoxins, and *Alternaria* toxins [18,19]. For example, *Penicillium expansum* causes the blue mold disease in apples and pears and is an important producer of patulin, leading to the contamination of fruit and their co-products (juices, mash, and freshly cut) [20,21,22]. 

In plant–pathogen dialogs, some mycotoxins are suspected to play a role in the modulation of defense signals transduced in the host [23]. For example, the deletion of the *Fusarium verticillioides SET1* gene reduced growth and fumonisin accumulation and limited the colonization process of the pathogen in maize stalks and kernels [24]. A *F. verticillioides FUG1* (*Fungal Unknown Gene 1*) deletion mutant produced fewer microconidia and no macroconidia. Following inoculation on kernels, *FUG1* mutants did not produce FB_1_ but produced more FB_2_ than in the wild type [25]. These studies demonstrated that toxins could support fungal virulence and the bypass of plant defenses [26].

Plant microbiomes are being explored to gain insight into the structure and function of microbial communities [27]. Previous studies on microbial interactions implicate fungal secondary metabolites as defense against other microorganisms [28,29,30]. Specifically, mycotoxins may have a role during interspecific competition related to the colonization of a habitat [31]. These interactions can be based on an exploitation competition, which is characterized by an extensive use of resources by one of the competing species and thereby limiting nutrient availability for competitors [32,33]. Alternatively, interference competition could occur if a competitor inhibits the growth and the spread of the competing species by the secretion of antibiotic metabolites in a given space [34,35]. Some microbial species are known to interact with or antagonize *Fusarium* species. These interactions modulate *Fusarium* toxin accumulation in host cellular compartments and decrease fungal virulence. For example, bacterial species such as *Pseudomonas fluorescens* can degrade and detoxify deoxynivalenol (DON) excreted by *Fusarium culmorum* [36]. Interestingly, fungi can also display similar behaviors, such as *Fusarium culmorum* and *Alternaria tenuissima.* Dual confrontations on wheat kernels showed *A. tenuissima* toxin production reduced to less than 5% while the biosynthesis of DON and zearalenone (ZEA) by *F. culmorum* increased 11- and 36-fold, respectively [37]. In vitro co-culture of *Fusarium graminearum* and *Aspergillus ochraceus* led to more accumulation of DON and ZEA during the early stages of interaction than observed in unchallenged cultures of *F. graminearum* [38]. Given their broad host range, *Fusarium* species can also interact with each other within a microbial community. For example, under in vitro conditions, *Fusarium poae* reduced the fumonisin production of *F. proliferatum* by more than 95% [39]. Similarly, *Fusarium temperatum* decreased to 32% the content of fumonisins accumulated by *F. verticillioides*. Beauvericin is considered a virulence factor for plants and is a growth inhibitor for the endophyte *Paraconiothyrium variabile* [40]. However, when interacting with *F. oxysporum*, *P. variabile* can metabolize beauvericin produced by *F. oxysporum* to protect its ecological niche [41]. These inter- and intra-species interactions emphasize the importance of considering fungi secondary metabolite production in the structure of microbial networks and the role of toxins in the pathogenesis [42]. 

The objective of the present study was to generate data on the behavior of interacting FCR pathogens under in vitro co-culture bioassays based on the hypothesis that a specific structure of the fungal–pathogenic community may favor FCR establishment. First, changes in fungal growth were monitored and the corresponding growth inhibition profiles were defined. Second, we characterized the influence of cross-challenged FCR pathogens on mycotoxin accumulation for 10 days. The data provide new insights into the microbial communication that may directly influence the occurrence of FCR disease and mycotoxin content in pineapple.

## 2. Results

### 2.1. Dynamic Evolution of Fungal Colonies

On Reunion Island, *F. ananatum*, *F. proliferatum*, *F. oxysporum*, and *T. stollii* were identified in diseased pineapple tissues and characterized as prevalent species of the fruit-associated mycobiome. Their ability to promote black spot symptoms was confirmed and all four fungal species are considered as FCR pathogens [6,43]. Searching for microbial interactions and chemical communication as potential drivers of the FCR pathogenic mycobiome (pathobiome) structure, one isolated strain of each species previously validated for Koch’s postulates was selected for co-culture bioassays [6,14]. 

According to single-conidia purification and molecular characterization, sequencing confirmed the identity of BP429 as *F. proliferatum*, BP369 as *F. oxysporum*, and BP462 as *T. stollii*, respectively, based on the targeted reference regions (Table S1).

**Figure 1:**
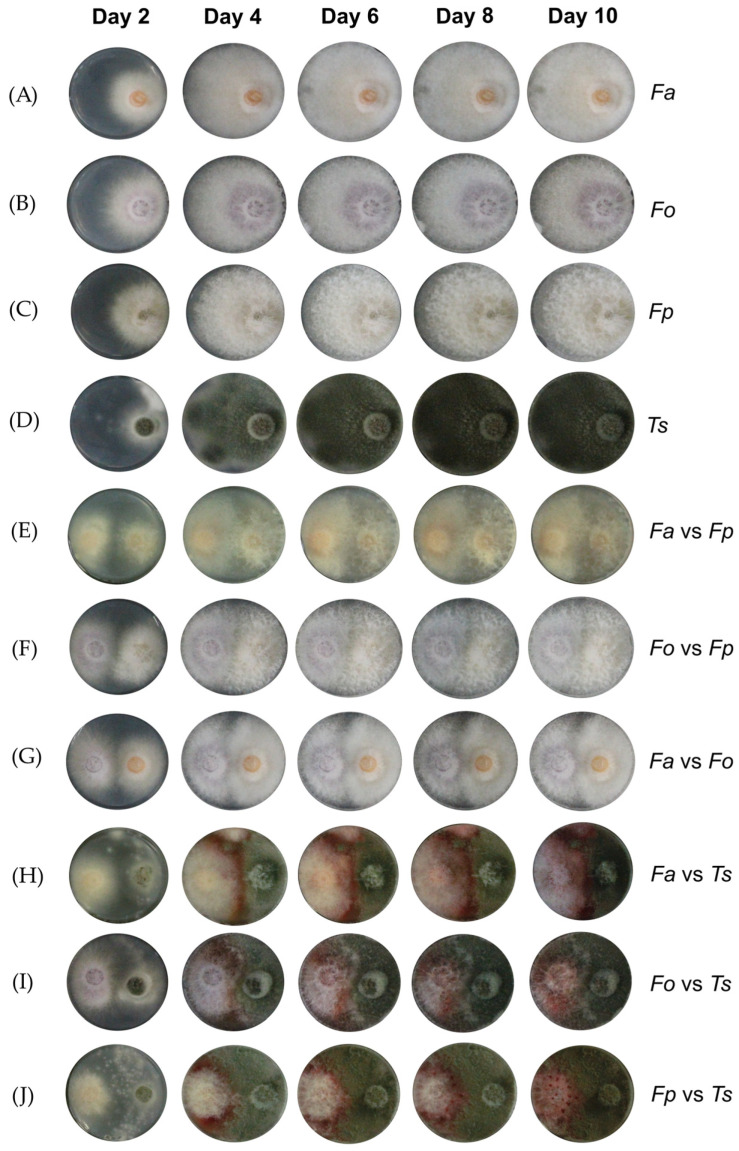
FCR pathogen colony aspects on PDA after 10 days of incubation in single culture of (**A**) *Fusarium ananatum* (*Fa*), (**B**) *Fusarium oxysporum* (*Fo*), (**C**) *Fusarium proliferatum* (*Fp*), and (**D**) *Talaromyces stollii* (*Ts*) or in condition of co-culture corresponding to (**E**) *F. ananatum* versus *F. proliferatum* (*Fa* vs. *Fp*), (**F**) *F. oxysporum* versus *F. proliferatum* (*Fo* vs. *Fp*), (**G**) *F. ananatum* versus *F. oxysporum* (*Fa* vs. *Fo*), (**H**) *F. ananatum* versus *T. stollii* (*Fa* vs. *Ts*), (**I**) *F. oxysporum* versus *T. stollii* (*Fo* vs. *Ts*), and (**J**) *F. proliferatum* versus *T. stollii* (*Fp* vs. *Ts*), well diameter = 2 cm.

Thereafter, both single and co-cultures were monitored at five time points to detect changes in mycelial growth and coloration. In single cultures, all studied *Fusarium* species showed a white mycelium after 2 days of incubation. From day 4, fungal colonies harbored pigmentation ranging from salmon pink (*F. ananatum*) to violet (*F. oxysporum*) and beige (*F. proliferatum*) on a PDA medium (Figure 1A–C and Figure S1A–C). *F. oxysporum* and *F. proliferatum* produced aerial mycelia, while *F. ananatum* was characterized by a short and dense mycelium. From this time on, no phenotypic variation was noticed until the end of the monitoring. Moreover, *Fusarium* species covered the entire surface of the wells within 6 days of incubation (Figure 1A–C). *T. stollii* showed a spotted white and light green colored mycelium at day 2 (Figure 1D and Figure S1D). *T. stollii* fully colonized the medium surface after 6 days of incubation, similarly to the *Fusarium* species. These colonies had a short and powdery dark green mycelium. Important changes in mycelial morphotypes were observed with the co-cultivation of the FCR pathogens. Following the culture of cross-challenged pathogenic *Fusarium* spp., the contact between mycelia was reported on day 4 and hyphae aerial profiles remained unchanged for the entire incubation period of *F. ananatum* versus *F. proliferatum*, *F. oxysporum* versus *F. proliferatum*, and *F. ananatum* versus *F. oxysporum* interaction conditions (Figure 1E–G and Figure S1E–G). Colony growth morphologies were similar to the single cultures of the corresponding species (Figure 1A–C,E–G and Figure S1A–C,E–G).

The most visible changes during co-culture evolution were observed between *Fusarium* spp. and *T. stollii* (Figure 1H–J). Growth antagonism was reported for all pairwise interactions combining *T. stollii* and *Fusarium* species. A zone line characterized by appearance of a red pigmentation occurred at the mycelial contact area for each pathogenic *Fusarium* spp. in the presence of *T. stollii* at day 4 and 6. At the subsequent time points, the intense red pigmentation spread all over the growth surface. In these conditions and from day 8, the *F. proliferatum* colony surface accumulated droplets of red exudate (Figure 1J). Co-culture of *F. ananatum* and *T. stollii* also led to the appearance of a red pigment. However, this was the only species combination with an inhibition zone line from day 4 to the end of the monitoring (Figure 1H). Finally, the red pigmentation highlighted over the set of *Fusarium*–*Talaromyces* co-cultures was localized and highly intense in the bottom side of the *Fusarium* colonies as shown in Figure S1 (Figure S1H–J).

### 2.2. Influence of FCR Pathogen Co-Cultivation on Growth and Inhibition Patterns

**Figure 2:**
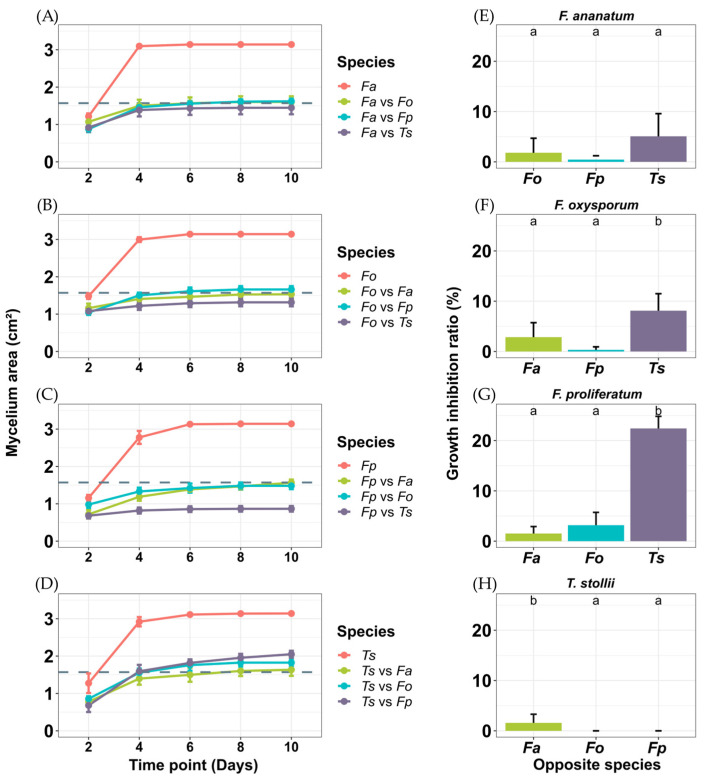
Influence of FCR pathogen interactions on colony growth parameters. (**A**–**D**) panels show fungal growth evolution over 10 days for co-cultures and corresponding single cultures. Gray dashed lines represent 50% of well area (1.57 cm^2^). (**E**–**H**) panels indicate associated growth inhibition ratio calculated from data at day 10 for each co-culture condition. (**A**,**E**); (**B**,**F**); (**C**,**G**) and (**D**,**H**) show growth profiles and inhibition potential on *F. ananatum*, *F. oxysporum*, *F. proliferatum*, and *T. stollii*, respectively. Vertical bars represent standard error of means (*n* = 9). Letters show significantly different inhibition ratios for each species following Tukey’s multiple comparison test at *p* < 0.05. *Fa*: *Fusarium ananatum*, *Fo*: *Fusarium oxysporum*, *Fp*: *Fusarium proliferatum*, and *Ts*: *Talaromyces stollii*.

Growth profiles of isolate strains were monitored over 10 days to track substrate prospection in single or co-cultures, and then define the associated inhibition rates. In single cultures, all four species grew rapidly until day 4 (Figure 2A–D). At day 6, the plate surface was completely colonized and slower growth was then observed until day 10. In co-cultures, the initial rapid growth phase occurred until day 6 for all experimental conditions. The data are consistent with antagonistic interactions in co-cultures of *Fusarium* spp. and *T. stollii*. Actually, *T. stollii* was the prevalent competitor with average inhibition ratios of 5%, 8%, and 22% against *F. ananatum*, *F. oxysporum*, and *F. proliferatum*, respectively (Figure 2E–G).

*T. stollii* had a high inhibition effect on the growth of *F. oxysporum* as compared to *F. ananatum* (*p* = 0.004) or *F. proliferatum* (*p* < 0.001; Figure 2F). The growth of *F. proliferatum* was also significantly reduced by *T. stollii* compared to co-cultures with *F. ananatum* (*p* < 0.001) and *F. oxysporum* (*p* < 0.001; Figure 2G). Interestingly, no significant difference was observed between inhibition potentials of *F. proliferatum*, *F. oxysporum*, and *T. stollii* on the mycelial growth of *F. ananatum*. Thus, all *Fusarium*–*Fusarium* co-cultures resulted in the equal occupation of the wells corresponding to half the surface (area of 1.57 cm^2^), indicating a low if not absent inhibition effect or competition (gray dashed line on Figure 2A–C). In contrast with the other *Fusarium* species, *F. ananatum* presented a slight, but significant, reduction in *T. stollii* growth with an inhibition by about 2% of the medium coverage compared to co-cultures with *F. oxysporum* (*p* = 0.02) and *F. proliferatum* (*p* = 0.02) (Figure 2H).

### 2.3. Modulation of Mycotoxin Production during Co-Culture Bioassays

**Figure 3:**
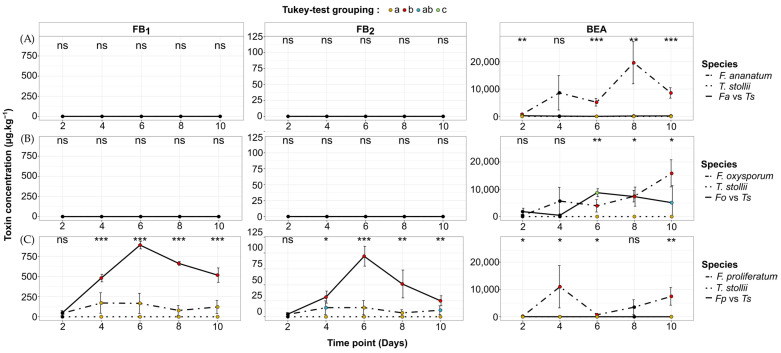
Dynamics of fumonisin B_1_ (FB_1_), fumonisin B_2_ (FB_2_), and beauvericin (BEA) during co-culture bioassays between *Fusarium* spp. and *Talaromyces stollii*. (**A**) *F. ananatum* versus *T. stollii* (*Fa* vs. *Ts*), (**B**) *F. oxysporum* vs. *T. stollii* (*Fo* vs. *Ts*), and (**C**) *F. proliferatum* versus *T. stollii* (*Fp* vs. *Ts*). Vertical bars represent standard deviation (*n* = 3). Differences between single and co-cultures were either significant at *p* < 0.05 (*), *p* < 0.01 (**), and *p* < 0.001 (***) or non-significant (ns) for each toxin and each time point. Dot colors indicate significant differences according to Tukey’s multiple comparison test at *p* < 0.05 and correspond to ‘a’ (yellow dots), ‘b’ (red dots), ‘ab’ (blue dots), and ‘c’ (green dots).

**Figure 4:**
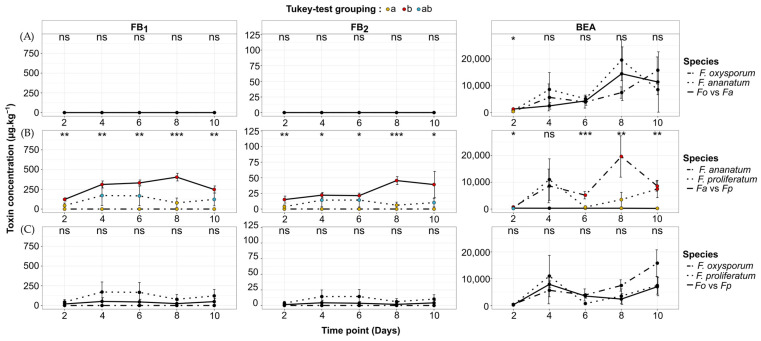
Dynamics of fumonisin B_1_ (FB_1_), fumonisin B_2_ (FB_2_), and beauvericin (BEA) during co-culture bioassays between *Fusarium* spp. reported as FCR pathogens. (**A**) *F. oxysporum* versus *F. ananatum* (*Fo* vs. *Fa*), (**B**) *F. ananatum* vs. *F. proliferatum* (*Fa* vs. *Fp*), and (**C**) *F. oxysporum* versus *F. proliferatum* (*Fo* vs. *Fp*). Vertical bars represent standard deviation (*n* = 3). Differences between single and co-cultures were either significant at *p* < 0.05 (*), *p* < 0.01 (**), and *p* < 0.001 (***) or non-significant (ns) for each toxin and each time point. Dot colors indicate significant differences according to Tukey’s multiple comparison test at *p* < 0.05 and correspond to ‘a’ (yellow dots), ‘b’ (red dots), and ‘ab’ (blue dots).

The *Fusarium* strains used in this study present contrasting toxigenic patterns in single cultures. In the sample set corresponding to *F. ananatum*, BEA was the only mycotoxin detected with a consistent maximum average concentration (av. ± sd) of 19,623 ± 7712 µg kg^−1^ recorded at day 8 (Figure 3A and Figure 4A,B). Similarly, *F. oxysporum* exclusively produced BEA quantified at its highest amount at 15,815 ± 4962 µg kg^−1^ at day 10 (Figure 3B and Figure 4A,C). The tested strain of *F. proliferatum* is a consistent producer of BEA with a peak of production at 10,998 ± 7741 µg kg^−1^ after 4 days of incubation. However, its biosynthesis patterns of FB_1_ and FB_2_ were slightly pronounced and correspond at their maximum to 171 ± 127 and 14 ± 11 µg kg^−1^, respectively (Figure 3C and Figure 4B,C). No toxins were identified or quantified from *T. stollii* single cultures even after 10 days of incubation (Figure 3A–C).

When compared to the single culture of *F. ananatum* (5173 ± 1384 µg kg^−1^), the co-culture of *F. ananatum* and *T. stollii* (28 ± 5 µg kg^−1^) significantly reduced the synthesis of BEA by 99.5% after 6 days of incubation (*p* < 0.001, Figure 3A). During the confrontation between *F. oxysporum* and *T. stollii* (8741 ± 1463 µg kg^−1^), the most significant change was also recorded at day 6 with a 2-fold increase in the BEA amount when compared to the *F. oxysporum* control condition (3945 ± 2260 µg kg^−1^, *p* = 0.02, Figure 3B). Across *F. proliferatum* versus *T. stollii*, BEA concentrations were significantly reduced when compared to the single culture of *F. proliferatum*. Indeed, BEA concentrations were close to the limit of detection (LOD) and limit of quantification (LOQ) (Figure 3C). Interestingly, the confrontation with *T. stollii* significantly raised both FB_1_ and FB_2_ productions by *F. proliferatum*. The most significant differences occurred at day 6 with a 5- and 7-fold increase in FB_1_ (*p* < 0.001) and FB_2_ (*p* < 0.001) concentrations in *F. proliferatum* versus *T. stollii* (FB_1_ = 890 ± 48 µg kg^−1^, FB_2_ = 95 ± 16 µg kg^−1^) when compared to the *F. proliferatum* single culture, respectively (FB_1_ = 167 ± 125 µg kg^−1^, FB_2_ = 14 ± 11 µg kg^−1^). Interestingly, this is the sole fungal confrontation for which, over several time points, significantly higher *Fusarium* toxin concentrations occurred by comparison to the production in single cultures. 

Interactions between *Fusarium*–FCR pathogen species also regulated mycotoxin content. At day 8, *F. proliferatum* versus *F. ananatum* co-culture (FB_1_ = 404 ± 48 µg kg^−1^, FB_2_ = 46 ± 6 µg kg^−1^) exhibited a 5- (*p* < 0.001) and 7-fold increase (*p* < 0.001) in FB_1_ and FB_2_ concentration in comparison to the *F. proliferatum* single culture (FB_1_ = 79 ± 61 µg kg^−1^, FB_2_ = 6 ± 5 µg kg^−1^, Figure 4B). By contrast, during this confrontation, the BEA amount dropped by 94%, 98%, and 97% at day 6, 8, and 10, respectively, according to the production recorded for the *F. ananatum* control condition (*p* ≤ 0.007). At day 2 of the *F. ananatum* versus *F. oxysporum* confrontation, an increase by 73% of BEA content was recorded (1327 ± 86 µg kg^−1^) against 767 ± 300 µg kg^−1^ synthetized by *F. ananatum* in the single culture (*p* = 0.01, Figure 4A). The subsequent time points demonstrated no significant change in BEA production for this co-culture condition. Finally, the interaction between *F. oxysporum* and *F. proliferatum* showed no significant differences in toxin production over the 10-day monitoring compared to the corresponding single cultures (Figure 4C).

## 3. Discussion

Exploring microbial interactions is crucial for the definition of the host–pathogens dialog especially in a multi-partite pathosystem such as pineapple FCR. Previously, species belonging to the *Fusarium fujikuroi* (FFSC) [44] and *Talaromyces purpureogenus* species complexes [45] as well as *F. oxysporum* were identified as the main risk of FCR occurrence in pineapple. Considering the relative abundance of species that composed the mycobiomes of healthy and diseased fruitlets, *T. stollii* corresponded to the third most frequent species, *F. proliferatum* and *F. ananatum* being the most abundant. *T. stollii* occurrence was 2-fold higher in diseased tissues than in healthy ones [6]. Thus, specific structure(s) of the pathobiome were hypothesized to determine the balance between healthy and diseased pineapple fruitlets. Considering the known toxinogenicity of *Fusarium* species, the impact of such interactions on the mycotoxin production in pineapple needed to be investigated to estimate potential consumer health problems. 

The approach by co-culture of fungal pathogens highlighted important changes in the evolution of mycelial growth. First, the growth curves and the relative inhibition ratios show that *T. stollii* is a major competitor of *Fusarium* species in the FCR complex, with the highest inhibitory effect on the growth and colonization of the three *Fusarium* species assayed. In fact, similar antagonistic patterns were observed following the challenged cultivation of *F. proliferatum* and *F. oxysporum* with *T. stollii*, suggesting an interference competition beneficial to *T. stollii*. By comparison, the co-cultivation of *F. ananatum* and *T. stollii* differs with the presence of an inhibition zone as previously defined by Bertrand et al. [46]. Growth patterns showed that *F. ananatum* partially limited the colonization of the culture medium surface by *T. stollii*. This is the only *Fusarium* species that, although being inhibited, succeeded in challenging *T. stollii*. The underlying mechanism remains to be elucidated. 

These results are consistent with those of Losada et al., which demonstrated that secondary metabolites are critical determinants of fungal competitive fitness [47]. Interestingly, the main growth inhibition ratio obtained from *T. stollii* is used against *F. proliferatum*, which was previously described as the most abundant species in relation to the proximal environment of pineapple plots [6]. The confrontation patterns facilitate the exploitation of resources from an artificial medium by *T. stollii* and suggested that its biomass may be highly abundant in the blossom cup of fruitlets. *Fusarium* species would then be restricted in host tissues in the form of conidia but remain detectable by classical microbiological approaches. Potential strategies of germination inhibition or mycelial proliferation delays still need to be elucidated. These antagonistic interactions are associated with a red pigmentation that could result from a pigment secretion. Particularly, when *Fusarium* species were confronted with *T. stollii*, the appearance of a red pigment was noticeable from day 4. Across the single cultures of the four species assayed, only a spotted red pigmentation was observed from the bottom of *T. stollii* wells (Figure S1D). This suggests that the expansion of a red coloration specifically resulted from the antagonistic interaction between *Fusarium* spp. and *T. stollii*. Fungi are widely known for their ability to produce bioactive compounds that support an interconnecting microbial network within an ecological niche [48,49]. Amongst the diverse secondary metabolites accumulated by fungi, some pigments were described for having antimicrobial, antifungal, antiviral, and other biological activities [50]. Although *T. stollii* was not previously studied in confrontation procedures, data are available for other *Talaromyces* species such as *T. purpureogenus* and *T. amestolkiae*, producing and diffusing red azaphilone polyketide pigments (‘Monascus pigments’) [51]. Alternatively, in vitro cultures of *F. fujikuroi*, *F. proliferatum*, and *F. oxysporum* can be associated with the excretion of pigments such as bikaverin, also defined as a mycotoxin [52,53,54]. *Fusarium* species are also major producers of secondary red-colored metabolites such as bostrycoidin, norjavanicin, and 8-O-methylfusarubin under conditions of environmental stress [55,56]. Thus, under antagonistic conditions, FCR pathogens, *Fusarium* species, could produce red pigments to manage their competitors. This conclusion is consistent with the study of Son et al., which demonstrated the efficient biocontrol activity of bikaverin and fusaric acid produced by *F. oxysporum* upon growth and development of *Phytophtora infestans* [57]. The red exudate on the mycelial surface of *F. proliferatum* after 8 days of co-cultivation with *T. stollii* could assign the red pigment biosynthesis to *Fusarium* species. In spite of the multiple mycotoxin standards used in this study (see Section Standard Solution), this specific secondary metabolite was not identified. This would help in defining the ecological balance between interference and exploitative competition [32,33,34,35]. This red exudate could also support an active detoxification of mycelium to reduce toxicity of reaction products released from the metabolization of compounds from the antagonist [58]. Therefore, we cannot conclude that this compound has a key role in the pathobiome structure and de facto in the occurrence of FCR. This still remains to be elucidated.

FB_1_, FB_2_, and BEA were the only identifiable and quantifiable mycotoxin compounds in our samples. In accordance with previous studies, the *Fusarium* strains isolated from diseased pineapple grown in Reunion Island can produce FB_1_, FB_2_, and BEA. Although not included in the set of mycotoxins searched for this study, the excretion of fumonisin B_3_ and moniliformin by pineapple-associated *Fusarium* spp. is also reported describing the wide toxigenic spectrum of these species [13,14,59,60]. Therefore, screening of FB_3_ and MON seems necessary for future studies on this ecological context. The combination of all studies demonstrate that pineapple fruit constitutes a paradigm to study *Fusarium* toxins contaminating fresh fruits. Mycotoxin risks associated with *Fusarium* species are mainly reported for cereals with less data focusing on fruit and vegetable productions. The study of FCR pathogen toxigenicity across in vitro confrontation demonstrated that the patterns of fumonisins and beauvericin production were drastically affected by fungal pairwise interactions. While the BEA content dropped in three of the six co-culture conditions tested, FB_1_ and FB_2_ produced by *F. proliferatum* rose significantly when challenged by *T. stollii* or *F. ananatum*. This increase highlighted that the chemical dialog is species-dependent [61]. Future studies are needed to determine whether the drop in beauvericin contents in species interactions results from a lack of biosynthesis or a detoxification process as reported for competition between *Paraconiothyrium variabile* and *F. oxysporum* [30]. Interestingly, most of the significant changes in mycotoxin concentrations occurred after six (or eight) days of interaction. This timing is consistent with the growth profiles observed in single cultures where the studied species fully colonized the medium surface within 6 days. Thus, fungal species development appeared to be regulated by the competitor. This was already observed in *Aspergillus fumigatus* for which the switch from vegetative growth to conidiation is regulated by phenazine-derived metabolites from *Pseudomonas aeruginosa* [62]. 

In the present study, we showed that *T. stollii* can compete with *Fusarium* species by reducing their growth and/or toxin production. These results are consistent with those of Camardo Leggieri et al., who found that the in vitro co-cultivation of *F. verticillioides* and *Aspergillus flavus* resulted in significant reductions in colony diameter. Moreover, the production of aflatoxin B_1_ and fumonisins B_1_ and B_2_ was significantly affected by culture conditions (single or dual) and the temperature of incubation [63]. Since mycotoxins can be considered in some cases as virulence factors, such changes could decrease the pathogenicity of *F. proliferatum*, *F. ananatum*, and *F. oxysporum*, thus enabling pineapple infection escape. Similarly, the co-culture of *F. verticillioides* with *Ustilago maydis* resulted in the secretion of fusaric acid by *F. verticillioides* that inhibited the growth of *U. maydis* and significantly decreased Corn Smut disease symptoms [64,65,66]. The molecular and biochemical mechanisms that characterize the FCR pathosystem also depend on the biomass of each protagonist. Thus, a low amount of *T. stollii* biomass may result in only the weak inhibition of *Fusarium* species. As a consequence, host tissues are colonized together with an increase in BEA accumulation by *F. ananatum*, *F. proliferatum*, and/or *F. oxysporum*. If toxins act as virulence factors, they could promote host susceptibility and disease expression [67]. Such a hypothesis is consistent with the levels of FB_1_ and BEA being higher in infected fruitlets than in healthy ones [14]. Fumonisins can act as a systemic signal with the accumulation of FB_1_ in maize leaf during root colonization by *F. verticillioides* [68]. Spatial and temporal patterns of pathogen species contamination are also important. Fungal spread is defined by landscape contextualization (i.e., configuration of host in its environment, proximal crops) and meteorological factors (i.e., wind, temperatures, and hygrometry) resulting in asynchronous species dispersions [69,70]. Environmental factors are also crucial, determining both plant resistance and fungal growth and virulence [71]. Thus, FCR occurrence could be supported by a specific temporal sequence of contamination. This sequence of fungal progression in the blossom cup probably determines, through the modulation of pathogen interactions, the growth of fungal biomass and defines the future pineapple resistance or susceptibility. This process was previously proposed for *Gibberella* and *Fusarium* ear rots of maize by demonstrating that infection partly relied on the combination of competitive and facilitative interactions between *F. graminearum* and *F. verticillioides* pathogens [72]. Moreover, *Fusarium* pathogens are well known for their ability to spread over fruit crops via irrigation water or wind [73,74]. Thus, initial contamination by one of the *Fusarium* species could result in elevated amounts of mycotoxins and, once again, in the susceptibility of pineapple fruit to FCR disease. Environmental conditions may significantly influence mycotoxigenic potentials of FCR pathogens especially in Reunion Island where production areas of the ‘Queen Victoria’ pineapple cultivar are characterized by an important diversity of microclimates [17]. Actually, pineapple presents important pH variations within the same infructescence due to the gradual maturation (from basal section to upper section) corresponding to the shell’s color [43]. For a mature infructescence, the mean pH of pineapple cv. Queen Victoria grown in Reunion Island is 3.4 [75]. Therefore, future studies are needed to determine the influence of pH on the toxigenicity of interacting FCR pathogens. In addition to the economic loss, FCR could be a hazard for human health in derivative products such as juice or canned pineapple. 

In Reunion Island, *F. ananatum* and *F. proliferatum* were identified as prevalent species in accordance with the numerous production areas present on the island [17]. When challenged by *T. stollii*, the toxin production profile of *F. proliferatum* differed from other *Fusarium* species–*T. stollii* combinations. Even if growth rates were the most impacted by *T. stollii* competition (growth inhibition of 22%), *F. proliferatum* still produced similar or higher levels of FB_1_ and FB_2_ over the 10 days of monitoring. *F. ananatum* was the only studied *Fusarium* species that could limit the growth of *T. stollii*. Thus, in interactions with *T. stollii*, *F. proliferatum* was the only FCR pathogen capable of partially preserving its mycotoxigenic potential while *F. ananatum* was the main growth challenger. In vivo, these two *Fusarium* species could combine to limit *T. stollii* but with an impact on quality due to their mycotoxin production. This hypothesis is in concordance with their respective distribution and abundance profiles across Reunion Island. These data demonstrated that interspecific interactions within the fungal community constitute key factors for the understanding of FCR etiology.

## 4. Conclusions

Our study provides a novel hypothesis on interspecific competition between *F. proliferatum*, *F. ananatum*, *F. oxysporum*, and *T. stollii*, the four fungal pathogens recently described as contributors to FCR epidemics in pineapple. We found that *T. stollii* is the main antagonist within the FCR fungal pathobiome. It appears that regulatory mechanisms of colonization profiles specifically rely on interactions between *T. stollii* and *Fusarium* FCR pathogens. This could therefore constitute a key determinant of biomass production in host tissues. Identifying the influence of temporal and environmental factors on fungal virulence would help more precisely define the scenario of FCR expression. Data are also necessary for deciphering the molecular crosstalk stimulating the biosynthesis of fumonisins B_1_ and B_2_ during interactions between FCR pathogenic species. Defining the modulations of a fungal network could contribute to the development of accurate prediction and prevention models. With climate change, such ecological and toxigenic mapping would promote effective risk management for food and feed safety. 

## 5. Materials and Methods

### 5.1. Microbial Material

#### 5.1.1. Fungal Strains

Four fungal strains previously isolated from black spots (naturally diseased fruitlets) of pineapple grown in Reunion Island were selected for their pathogenicity in FCR: *Fusarium ananatum* (clp001), *Fusarium proliferatum* (BP429), *Fusarium oxysporum* (BP369), and *Talaromyces stollii* (BP462) [6,14]. The following procedure was performed on strains BP429, BP369, and BP462. The fungal strain clp001 was obtained by Barral et al. [14]. 

For identification, single conidia isolation was carried out for each strain by cultivation on Malt agar (pH 5.5, BD Difco^TM^, Le Pont-de-Claix, France) for seven days at 27 °C in the dark. Consequently, a conidia solution was prepared by adding 2 mL of Saline Peptone Water (Biokar diagnostic, Solabia, Allonne, France) to the Petri dish and gently scratching the mycelium surface with a sterile spreader. From the conidia suspension, 1 mL was transferred into sterile cryovials with the addition of an equal volume of 40% glycerol (Honeywell Riedel-de-Haën™, Seelze, Germany) and stored at −80 °C in the fungi collection at CIRAD Ligne-Paradis, La Réunion, France. Another volume of 750 µL was recovered for the molecular identification procedure and stored at −20 °C.

##### DNA Extraction

For each fungal strain, 500 µL of conidia suspension was collected in 2 mL sterile tubes and centrifuged at 14,000× *g* for 2 min. DNA extractions were performed on the biomass pellets with the FastDNA SPIN kit and the FastPrep-24 Instrument (MP Biomedicals^®^, Illkirch, France) using Lysing Matrix A and Lysis Buffer CLS-Y in accordance with the manufacturer’s instructions.

##### Molecular Characterization

For *Fusarium* sp. isolates, the Translation Elongation Factor-1α (TEF-1α), Internal Transcribed Spacer (ITS), and β-tubulin reference gene regions were amplified by PCR while ITS and β-tubulin regions were targeted for *Talaromyces stollii*. Primer sequences used in this study are shown in Table 1. 

PCR amplification reactions were performed in a final volume of 50 µL containing 0.3 µM of each primer, all the deoxyribonucleotide triphosphates (dNTPs) at 0.2 mM, 2 mM MgCl_2_, 10 µL of 5X GoTaq Flexi buffer, 1.25 U of the GoTaq^®^ G2 Flexi DNA polymerase (Promega, Charbonnières-les-Bains, France), and 2 µL of the fungal DNA extract. Conditions for the PCR amplification of the TEF-1α region were an initial denaturation at 95 °C for 3 min; 33 cycles at 95 °C for 30 s, 55 °C for 30 s, and 72 °C for 1 min; and a final extension stage of 5 min at 72 °C. Similarly, PCR amplification reactions for the β-tubulin region were established as follows: an initial denaturation at 95 °C for 2 min; 30 cycles at 95 °C for 30 s, 62 °C for 30 s, and 72 °C for 30 s; and a final extension at 72 °C for 5 min. PCR amplification reactions for the ITS region were carried out as follows: an initial denaturation at 95 °C for 2 min; 40 cycles at 95 °C for 15 s, 53 °C for 30 s, and 72 °C for 45 s; and a final extension at 72 °C for 5 min. The PCR reactions were performed in a thermal cycler (GS1, G-Storm, Braintree, UK). PCR products were analyzed with the Qiaxcel^®^ Advanced System (Qiagen, Hilden, Germany) using size markers, 250 bp–4 kb. PCR products were sent to Macrogen (Amsterdam, the Netherlands) for purification and sequencing. The DNA sequences obtained were aligned with SnapGene v5.0 software, and identification was performed using a BLASTn similarity search. Sequences having a percentage of identity of at least 98% and those with the lowest E-values were considered as belonging to the same species.

#### 5.1.2. Co-Culture Bioassays

Prior to co-culture experiments, pre-cultures of each strain were grown in triplicate in Petri dishes on potato dextrose agar (PDA, pH 5.6, VWR International, Fontenay-Sous-Bois, France) for 9 days at 27 °C in the dark.

The procedure was adapted from Bertrand et al. [82], where 2 mL of PDA was distributed into 12-well culture plates with an internal diameter of 2 cm (ThermoFisher Scientific, Illkirch, France). In co-culture assays, strains were challenged as follows: *F. ananatum* (clp001) versus *F. oxysporum* (BP369), *F. ananatum* (clp001) versus *F. proliferatum* (BP429), *F. oxysporum* (BP369) versus *F. proliferatum* (BP429), *F. ananatum* (clp001) versus *T. stollii* (BP462), *F. oxysporum* (BP369) versus *T. stollii* (BP462), and *F. proliferatum* (BP429) versus *T. stollii* (BP462). Wells were inoculated with agar plugs of a 2 mm diameter obtained from pre-cultures and removed from the center of the colonies. For the co-culture condition, agar plugs were deposited at, respectively, 1- and 3-quarter positions along the well diameter (i.e., at 0.5 cm distance from the margin of the well). Control wells were inoculated with single strains. Both confronted and control conditions were performed in triplicate. Thus, a plate was devoted to one co-culture condition and contained 3 wells of single cultures of both strain A and B, 3 wells of co-culture of strain A versus strain B, and 3 non-inoculated wells (Figure S2). Incubation was performed for 2, 4, 6, 8, or 10 days at 27 °C in the dark. At each time point, the three replicate wells were sampled (mycelium and growth medium were collected), pooled, frozen in liquid nitrogen, and stored at −80 °C for further analyses. Three technical replicates were performed corresponding to a total of 90 plates (1 plate per co-culture condition and per time point, 6 co-culture conditions, 5 time points, and 3 biological replicates).

### 5.2. Mycotoxin Identification and Quantification

#### 5.2.1. Extraction Procedure

The contents of the three pooled wells were ground at 5 m s^−1^ for 15 s with two ceramic beads using the FastPrep-24 Instrument (MP Biomedicals^®^, Illkirch, France). From the homogenized matrix, 2.5 g was weighed and 14 mL of acetonitrile/H_2_O/acetic acid (79:5:1, *v*/*v*/*v*) added. Samples were vortexed and placed in a shaking incubator for 30 min at 200 rpm (Gerhardt Analytical Systems, Königswinter, Germany). After 3 min of centrifugation at 1000× *g*, 1 mL of the supernatant was transferred to an amber glass vial and dried at 40 °C under nitrogen flow. After evaporation, 1 mL of a 99.5/0.5% (*v*/*v*) H_2_O/acetic acid solution of an internal standard (IS) mix (see Section Standard Solution) was added and the solution was filtered with a 0.45 μm PTFE (polytetrafluoroethylene) syringe filter (ClearLine, Issy-les-Moulineaux, France). The filtrate was used for the determination of mycotoxin content.

#### 5.2.2. Tandem Mass Spectrometry Assay

##### UHPLC MS/MS Analysis

For all samples, mycotoxins were detected and confirmed by Ultra High-Performance Liquid Chromatography (UHPLC, Shimadzu, Tokyo, Japan) coupled with a mass spectrometer (8040, Shimadzu, Tokyo, Japan) and quantified by isotope-labeled internal standards (ISs) following the protocol of Capodanno et al. and Moreau and Levi [83,84]. The data were analyzed using LabSolutions software (v5.91/2017, Shimadzu, Tokyo, Japan, 2017). The limit of detection (LOD) and limit of quantification (LOQ) were, respectively, 0.03/0.1 ng mL^−1^ for FB_1_, 0.01/0.05 ng mL^−1^ for FB_2_, and 0.05/0.15 ng mL^−1^ for BEA.

##### UHPLC Conditions

Chromatographic separation was carried out with a Kinetex 2.6 μm C18 100A 50 × 2.1 mm ID column (Phenomenex, Torrance, CA, USA). The column temperature was maintained at 50 °C and injection was performed with a volume of 50 μL. The mobile phase A consisted of 99.5% water and 0.5% acetic acid (HPLC MS Grade, Sigma-Aldrich, Darmstadt, Germany). Mobile phase B consisted of 99.5% isopropanol (HPLC MS Grade, Biosolve, Dieuze, France) and 0.5% acetic acid (HPLC MS Grade, Sigma-Aldrich, Darmstadt, Germany). A mobile phase gradient program was started at 90% A (0.01 min), 45% A at 1.5 min, 15% A at 3.5 min, 20% A at 4 min, 98% A at 4.01 min, and finally 98% A at 11 min. The flow rate was 0.4 mL min^−1^.

##### MS/MS Conditions

The mass spectrometer was operated in electrospray ionization, and positive (ESI+) and negative (ESI−) ionization modes. Two multiple reaction monitoring (MRM) transitions for each analyte were monitored for Quantification (Q) and Qualification (q). The parameters of the mass spectrometer were as follows: desolvation line—250 °C; heater block—400 °C; nebulizing gas—3 L min^−1^; drying gas—15 L min^−1^; CID gas pressure—270 kPa (Argon).

##### Standard Solution

The certified isotope-labeled standard solutions were purchased from Romer Labs Diagnostic GmbH (Tulln, Austria). The certified standard solutions of aflatoxins (B_1_, B_2_, G_1_, and G_2_), fumonisins (B_1_ and B_2_), trichothecenes (DON, nivalenol, 15ADON, 3ADON, HT-2 toxin, T2 toxin), zearalenone (ZEA), citrinin, ochratoxin A (OTA), and beauvericin (BEA) were purchased from R-Biopharm (Saint-Didier-au-Mont-d’Or, France). A composite standard and internal standard (IS) working solution of all the mycotoxins was prepared by dissolving appropriate volumes of each compound in a mobile phase A: 99.5% water and 0.5% acetic acid. Stock solutions were diluted with mobile phase A, to obtain working solutions for calibration. All solutions were stored at −20 °C in amber glass vials and darkness before use.

### 5.3. Computational Analysis

#### 5.3.1. Calculations of Areas and Inhibition Ratios

The colony growth rates were determined by an image analysis with the ImageJ software (v.1.53.a, U.S. National Institutes of Health, Bethesda, MD, USA, 2020) considering the mycelium area in each well for both single and co-cultures [85]. 

Patterns of inhibition growth were calculated for each co-culture condition after 10 days of incubation with the formula

I%=[[(R1−R2)/R1]×100]−50

where *R*1 corresponds to the mycelial area of the studied species in the control condition (single culture) and *R*2 to the mycelial area of the studied species in co-culture [86].

#### 5.3.2. Statistical Analysis

Data were analyzed with programs available in R v4.0.3 (R Core Team, 2020). First, significant differences between inhibition ratios for each studied species in the condition of co-culture were tested with a Deviance test and a Tukey’s multiple comparison test. 

The effect of species culture conditions on toxin accumulation was tested for each identified mycotoxin and at each time point with a Deviance test on independent generalized linear models. Multiple comparisons between means of mycotoxin concentrations at the different incubation times and for the different culture conditions were tested with the Tukey’s test.

## Figures and Tables

**Table 1:** DNA primer sequences (forward and reverse) used for fungal strain identification.

Sequence ID of Primer Pair	Target Locus	Forward Sequence (5′→3′)	Reverse Sequence (5′→3′)	Product Length (bp)	References
ef1/ef2	Translation Elongation Factor-1α	ATGGGTAAGGAAGACAAGAC	GGAAGTACCAGTGATCATGTT	380–680	[76,77,78]
Bt2a/Bt2b	β-Tubulin	GGTAACCAAATCGGTGCTGCTTTC	ACCCTCAGTGTAGTGACCCTTGGC	250–500	[79]
ITS1F/ITS4	Entire Internal Transcribed Spacer	CTTGGTCATTTAGAGGAAGTAA	TCCTCCGCTTATTGATATGC	600–800	[80,81]

## Data Availability

The data presented in this study are available on request from the corresponding authors.

## Supplementary Materials

The following supporting information can be downloaded at: https://www.mdpi.com/article/10.3390/toxins16080344/s1, Figure S1: FCR pathogen colony aspects on PDA view from bottom of culture plates after 10 days of incubation in single culture of (A) *Fusarium ananatum* (*Fa*), (B) *Fusarium oxysporum* (*Fo*), (C) *Fusarium proliferatum* (*Fp*), and (D) *Talaromyces stollii* (*Ts*) or in condition of co-culture corresponding to (E) *F. ananatum* versus *F. proliferatum* (*Fa* vs. *Fp*), (F) *F. oxysporum* versus *F. proliferatum* (*Fo* vs. *Fp*), (G) *F. ananatum* versus *F. oxysporum* (*Fa* vs. *Fo*), (H) *F. ananatum* versus *T. stollii* (*Fa* vs. *Ts*), (I) *F. oxysporum* versus *T. stollii* (*Fo* vs. *Ts*), and (J) *F. proliferatum* versus *T. stollii* (*Fp* vs. *Ts*), well diameter = 2 cm; Figure S2: Co-culture procedure performed with fungal species defined as FCR pathogens of pineapple on 12-well culture plates. 1,2: single cultures, 3: co-cultures, 4: non-inoculated wells; Table S1: Identification of fungal species isolated from naturally infected pineapple fruitlets and selected for co-culture bioassays.

## References

[1] Gu H., Zhan R.L., Zhang L.B., Gong D.Q., Jia Z.W. (2015). First Report of Fusarium Ananatum Causing Pineapple Fruitlet Core Rot in China. Plant Dis..

[2] Jacobs A., Wyk P.S., Marasas W.F.O., Wingfield B.D., Wingfield M.J., Coutinho T.A. (2010). Fusarium Ananatum Sp. Nov. in the Gibberella Fujikuroi Species Complex from Pineapples with Fruit Rot in South Africa. Fungal Biol..

[3] Barral B., Chillet M., Minier J., Léchaudel M., Schorr-Galindo S. (2017). Evaluating the response to Fusarium ananatum inoculation and antifungal activity of phenolic acids in pineapple. Fungal Biol..

[4] Barral B., Chillet M., Léchaudel M., Lartaud M., Verdeil J.-L., Conéjéro G. (2019). An Imaging Approach to Identify Mechanisms of Resistance to Pineapple Fruitlet Core Rot. Front. Plant Sci..

[5] Mourichon X. (1997). Pineapple Fruitlet Core Rot (Black Spot) and Leathery Pocket: Review and Prospects. Acta Hortic..

[6] Vignassa M., Meile J.-C., Chiroleu F., Soria C., Leneveu-Jenvrin C., Schorr-Galindo S. (2021). Pineapple Mycobiome Related to Fruitlet Core Rot Occurrence and the Influence of Fungal Species Dispersion Patterns. JoF.

[7] Li Z.F., He C.L., Wang Y., Li M.J., Dai Y.J., Wang T. (2016). Enhancement of trichothecene mycotoxins of Fusarium oxysporum by ferulic acid aggravates oxidative damage in Rehmannia glutinosa Libosch. Sci. Rep..

[8] Gálvez L., Urbaniak M., Waśkiewicz A., Stępień Ł., Palmero D. (2017). *Fusarium proliferatum*—Causal Agent of Garlic Bulb Rot in Spain: Genetic Variability and Mycotoxin Production. Food Microbiol..

[9] Li T., Gong L., Jiang G., Wang Y., Gupta V.K., Qu H. (2017). Carbon sources influence fumonisin production in *Fusarium proliferatum*. Proteomics.

[10] Shao C., Xiang D., Wei H., Liu S., Yi G., Lyu S. (2020). Predicting Virulence of Fusarium Oxysporum f. Sp. Cubense Based on the Production of Mycotoxin Using a Linear Regression Model. Toxins.

[11] Hussein H.S., Brasel J.M. (2001). Toxicity, Metabolism, and Impact of Mycotoxins on Humans and Animals. Toxicology.

[12] European Food Safety Authority Mycotoxins. https://www.efsa.europa.eu/en/topics/topic/mycotoxins.

[13] Stępień Ł., Koczyk G., Waśkiewicz A. (2013). Diversity of Fusarium species and mycotoxins contaminating pineapple. J. Appl. Genet..

[14] Barral B., Chillet M., Doizy A., Grassi M., Ragot L., Léchaudel M. (2020). Diversity and Toxigenicity of Fungi That Cause Pineapple Fruitlet Core Rot. Toxins.

[15] Jackson L.S., Al-Taher F. (2008). Factors Affecting Mycotoxin Production in Fruits. Mycotoxins in Fruits and Vegetables.

[16] Kumar D., Barad S., Sionov E., Keller N., Prusky D. (2017). Does the Host Contribute to Modulation of Mycotoxin Production by Fruit Pathogens?. Toxins.

[17] Fournier P., Benneveau A., Hardy C., Chillet M., Léchaudel M. (2015). A Predictive Model Based on a Pluviothermic Index for Leathery Pocket and Fruitlet Core Rot of Pineapple cv. ‘Queen’. Eur. J. Plant Pathol..

[18] Moss M.O. (2008). Fungi, Quality and Safety Issues in Fresh Fruits and Vegetables. J. Appl. Microbiol..

[19] Fernández-Cruz M.L., Mansilla M.L., Tadeo J.L. (2010). Mycotoxins in Fruits and Their Processed Products: Analysis, Occurrence and Health Implications. J. Adv. Res..

[20] Spadaro D., Ciavorella A., Frati S., Garibaldi A., Gullino M.L. (2007). Incidence and Level of Patulin Contamination in Pure and Mixed Apple Juices Marketed in Italy. Food Control.

[21] Puel O., Galtier P., Oswald I. (2010). Biosynthesis and Toxicological Effects of Patulin. Toxins.

[22] Coton M., Bregier T., Poirier E., Debaets S., Arnich N., Coton E. (2020). Production and Migration of Patulin in Penicillium Expansum Molded Apples during Cold and Ambient Storage. Int. J. Food Microbiol..

[23] Diamond M., Reape T.J., Rocha O., Doyle S.M., Kacprzyk J., Doohan F.M. (2013). The Fusarium Mycotoxin Deoxynivalenol Can Inhibit Plant Apoptosis-like Programmed Cell Death. PLoS ONE.

[24] Gu Q., Tahir H., Zhang H., Huang H., Ji T., Sun X. (2017). Involvement of FvSet1 in Fumonisin B1 Biosynthesis, Vegetative Growth, Fungal Virulence, and Environmental Stress Responses in Fusarium Verticillioides. Toxins.

[25] Ridenour J.B., Bluhm B.H. (2017). The Novel Fungal-specific Gene FUG1 Has a Role in Pathogenicity and Fumonisin Biosynthesis in *Fusarium verticillioides*. Mol. Plant Pathol..

[26] Ma L.-J., Geiser D.M., Proctor R.H., Rooney A.P., O’Donnell K., Trail F. (2013). *Fusarium* Pathogenomics. Annu. Rev. Microbiol..

[27] Banerjee S., Schlaeppi K., Heijden M.G.A. (2018). Keystone Taxa as Drivers of Microbiome Structure and Functioning. Nat. Rev. Microbiol..

[28] Vinale F., Marra R., Scala F., Ghisalberti E.L., Lorito M., Sivasithamparam K. (2006). Major Secondary Metabolites Produced by Two Commercial Trichoderma Strains Active against Different Phytopathogens. Lett. Appl. Microbiol..

[29] You F., Han T., Wu J., Huang B., Qin L. (2009). Antifungal Secondary Metabolites from Endophytic *Verticillium* sp.. Biochem. Syst. Ecol..

[30] Combès A., Ndoye I., Bance C., Bruzaud J., Djediat C., Dupont J. (2012). Chemical Communication between the Endophytic Fungus Paraconiothyrium Variabile and the Phytopathogen Fusarium Oxysporum. PLoS ONE.

[31] Knowles S.L., Raja H.A., Wright A.J., Lee A.M.L., Caesar L.K., Cech N.B. (2019). Mapping the Fungal Battlefield: Using in Situ Chemistry and Deletion Mutants to Monitor Interspecific Chemical Interactions between Fungi. Front. Microbiol..

[32] Sarrocco S., Mauro A., Battilani P. (2019). Use of Competitive Filamentous Fungi as an Alternative Approach for Mycotoxin Risk Reduction in Staple Cereals: State of Art and Future Perspectives. Toxins.

[33] Pellan L., Durand N., Martinez V., Fontana A., Schorr-Galindo S., Strub C. (2020). Commercial biocontrol agents reveal contrasting comportments against two mycotoxigenic fungi in cereals: *Fusarium graminearum* and *Fusarium verticillioides*. Toxins.

[34] Wicklow D.T. (1992). The Fungal Community: Its Organization and Role in the Ecosystem. Supplied by U.S. Department of Agriculture, National Center 624 for Agricultural Utilization Research.

[35] Ghoul M., Mitri S. (2016). The Ecology and Evolution of Microbial Competition. Trends Microbiol..

[36] Khan M.R., Doohan F.M. (2009). Bacterium-Mediated Control of Fusarium Head Blight Disease of Wheat and Barley and Associated Mycotoxin Contamination of Grain. Biol. Control.

[37] Müller M.E.H., Steier I., Köppen R., Siegel D., Proske M., Korn U. (2012). Cocultivation of Phytopathogenic Fusarium and Alternaria Strains Affects Fungal Growth and Mycotoxin Production. J. Appl. Microbiol..

[38] Milles J., Krämer J., Prange A. (2007). In vitro competitive interactions of *Fusarium graminearum* with *Aspergillus ochraceus* and *Penicillium verrucosum* with regard to mycotoxin production. J. Food Agric. Environ..

[39] Ridout M.E., Godfrey B., Newcombe G. (2019). Effects of Antagonists on Mycotoxins of Seedborne *Fusarium* spp. in Sweet Corn. Toxins.

[40] López-Berges M.S., Hera C., Sulyok M., Schäfer K., Capilla J., Guarro J. (2013). The Velvet Complex Governs Mycotoxin Production and Virulence of *Fusarium oxysporum* on Plant and Mammalian Hosts: Velvet Governs Mycotoxins and Virulence in Fusarium. Mol. Microbiol..

[41] Bärenstrauch M., Mann S., Jacquemin C., Bibi S., Sylla O.-K., Baudouin E. (2020). Molecular Crosstalk between the Endophyte *Paraconiothyrium variabile* and the Phytopathogen *Fusarium oxysporum*—Modulation of Lipoxygenase Activity and Beauvericin Production during the Interaction. Fungal Genet. Biol..

[42] Marmann A., Aly A., Lin W., Wang B., Proksch P. (2014). Co-Cultivation—A Powerful Emerging Tool for Enhancing the Chemical Diversity of Microorganisms. Mari. Drugs.

[43] Barral B. (2017). Maladie des Taches Noires de L’ananas: Étude des Relations Hôte-Pathogène et Compréhension des Mécanismes Physiologiques de Résistance.

[44] Wigmann É.F., Behr J., Vogel R.F., Niessen L. (2019). MALDI-TOF MS Fingerprinting for Identification and Differentiation of Species within the *Fusarium fujikuroi* Species Complex. Appl. Microbiol. Biotechnol..

[45] Yilmaz N., Houbraken J., Hoekstra E.S., Frisvad J.C., Visagie C.M., Samson R.A. (2012). Delimitation and Characterisation of *Talaromyces purpurogenus* and Related Species. Pers.-Int. Mycol. J..

[46] Bertrand S., Schumpp O., Bohni N., Bujard A., Azzollini A., Monod M. (2013). Detection of Metabolite Induction in Fungal Co-Cultures on Solid Media by High-Throughput Differential Ultra-High Pressure Liquid Chromatography–Time-of-Flight Mass Spectrometry Fingerprinting. J. Chromatogr. A.

[47] Losada L., Ajayi O., Frisvad J.C., Yu J., Nierman W.C. (2009). Effect of Competition on the Production and Activity of Secondary Metabolites in *Aspergillus* Species. Med. Mycol..

[48] Fox E.M., Howlett B.J. (2008). Secondary Metabolism: Regulation and Role in Fungal Biology. Curr. Opin. Microbiol..

[49] Costa Souza P., Luiza Bim Grigoletto T., Moraes L., Abreu L.M., Henrique Souza Guimarães L., Santos C. (2016). Production and Chemical Characterization of Pigments in filamentous fungi. Microbiology.

[50] Sánchez-Muñoz S., Mariano-Silva G., Leite M.O., Mura F.B., Verma M.L., Silva S.S. (2020). Production of Fungal and Bacterial Pigments and Their Applications. Biotechnological Production of Bioactive Compounds.

[51] Yilmaz N., Visagie C.M., Houbraken J., Frisvad J.C., Samson R.A. (2014). Polyphasic taxonomy of the genus *Talaromyces*. Stud. Mycol..

[52] Lebeau J., Petit T., Clerc P., Dufossé L., Caro Y. (2019). Isolation of Two Novel Purple Naphthoquinone Pigments Concomitant with the Bioactive Red Bikaverin and Derivates Thereof Produced by *Fusarium oxysporum*. Biotechnol. Prog..

[53] Lebeau J., Petit T., Dufossé L., Caro Y. (2019). Putative Metabolic Pathway for the Bioproduction of Bikaverin and Intermediates Thereof in the Wild *Fusarium oxysporum* LCP531 Strain. AMB Express.

[54] Gasser K., Sulyok M., Spangl B., Krska R., Steinkellner S., Hage-Ahmed K. (2023). *Fusarium proliferatum* Secondary Metabolite Profile in Vitro Depends on the Origin of the Isolates and Is Clearly Reduced in Stored Garlic. Postharvest Biol. Technol..

[55] Studt L., Wiemann P., Kleigrewe K., Humpf H.-U., Tudzynski B. (2012). Biosynthesis of fusarubins accounts for pigmentation of *Fusarium fujikuroi* perithecia. Appl. Environ. Microbiol..

[56] Lagashetti A.C., Dufossé L., Singh S.K., Singh P.N. (2019). Fungal Pigments and Their Prospects in Different Industries. Microorganisms.

[57] Son S.W., Kim H.Y., Choi G.J., Lim H.K., Jang K.S., Lee S.O. (2008). Bikaverin and Fusaric Acid from *Fusarium oxysporum* Show Antioomycete Activity against *Phytophthora infestans*. J. Appl. Microbiol..

[58] Dullah S., Hazarika D.J., Goswami G., Borgohain T., Ghosh A., Barooah M., Bhattacharyya A., Boro R.C. (2021). Melanin Production and Laccase Mediated Oxidative Stress Alleviation during Fungal-Fungal Interaction among Basidiomycete Fungi. IMA Fungus.

[59] Stępień Ł., Waśkiewicz A. (2013). Sequence Divergence of the Enniatin Synthase Gene in Relation to Production of Beauvericin and Enniatins in *Fusarium* Species. Toxins.

[60] Ibrahim N.F., Mohd M.H., Mohamed Nor N.M.I., Zakaria L. (2020). Mycotoxigenic Potential of Fusarium Species Associated with Pineapple Diseases. Arch. Phytopathol. Plant Prot..

[61] Venkatesh N., Keller N.P. (2019). Mycotoxins in Conversation with Bacteria and Fungi. Front. Microbiol..

[62] Zheng H., Kim J., Liew M., Yan J.K., Herrera O., Bok J.W. (2015). Redox metabolites signal polymicrobial biofilm development via the NapA oxidative stress cascade in Aspergillus. Curr. Biol..

[63] Camardo Leggieri M., Giorni P., Pietri A., Battilani P. (2019). *Aspergillus flavus* and *Fusarium verticillioides* Interaction: Modeling the Impact on Mycotoxin Production. Front. Microbiol..

[64] Lee K., Pan J.J., May G. (2009). Endophytic Fusarium Verticillioides Reduces Disease Severity Caused by Ustilago Maydis on Maize. FEMS Microbiol. Lett..

[65] Rodriguez Estrada A.E., Hegeman A., Corby Kistler H., May G. (2011). *In Vitro* Interactions between *Fusarium verticillioides* and *Ustilago maydis* through Real-Time PCR and Metabolic Profiling. Fungal Genet. Biol..

[66] Jonkers W., Rodriguez Estrada A.E., Lee K., Breakspear A., May G., Kistler H.C. (2012). Metabolome and Transcriptome of the Interaction between *Ustilago maydis* and *Fusarium verticillioides In Vitro*. Appl. Environ. Microbiol..

[67] Perincherry L., Lalak-Kańczugowska J., Stępień Ł. (2019). Fusarium-Produced Mycotoxins in Plant-Pathogen Interactions. Toxins.

[68] Baldwin T.T., Zitomer N.C., Mitchell T.R., Zimeri A.-M., Bacon C.W., Riley R.T. (2014). Maize Seedling Blight Induced by *Fusarium verticillioides*: Accumulation of Fumonisin B_1_ in Leaves without Colonization of the Leaves. J. Agric. Food Chem..

[69] Parnell S., Gottwald T.R., Gilligan C.A., Cunniffe N.J., Bosch F. (2010). The effect of landscape pattern on the optimal eradication zone of an invading epidemic. Phytopathology®.

[70] Almaguer M., Aira M.-J., Rodríguez-Rajo F.J., Rojas T.I. (2014). Temporal Dynamics of Airborne Fungi in Havana (Cuba) during Dry and Rainy Seasons: Influence of Meteorological Parameters. Int. J. Biometeorol..

[71] Velásquez A.C., Castroverde C.D.M., He S.Y. (2018). Plant–Pathogen Warfare under Changing Climate Conditions. Curr. Biol..

[72] Picot A., Hourcade-Marcolla D., Barreau C., Pinson-Gadais L., Caron D., Richard-Forget F. (2012). Interactions between *Fusarium verticillioides* and *Fusarium graminearum* in Maize Ears and Consequences for Fungal Development and Mycotoxin Accumulation: *Fusarium* spp. Interactions in Maize Ears. Plant Pathol..

[73] Dita M., Barquero M., Heck D., Mizubuti E.S.G., Staver C.P. (2018). Fusarium Wilt of Banana: Current Knowledge on Epidemiology and Research Needs toward Sustainable Disease Management. Front. Plant Sci..

[74] Ou Y., Penton C.R., Geisen S., Shen Z., Sun Y., Lv N. (2019). Deciphering underlying drivers of disease suppressiveness against pathogenic *Fusarium oxysporum*. Front. Microbiol..

[75] Leneveu-Jenvrin C., Quentin B., Assemat S., Hoarau M., Meile J.-C., Remize F. (2020). Changes of Quality of Minimally-Processed Pineapple (*Ananas Comosus*, Var. ‘Queen Victoria’) during Cold Storage: Fungi in the Leading Role. Microorganisms.

[76] O’Donnell K., Cigelnik E., Nirenberg H.I. (1998). Molecular Systematics and Phylogeography of the *Gibberella fujikuroi* Species Complex. Mycologia.

[77] O’Donnell K., Kistler H.C., Cigelnik E., Ploetz R.C. (1998). Multiple Evolutionary Origins of the Fungus Causing Panama Disease of Banana: Concordant Evidence from Nuclear and Mitochondrial Gene Genealogies. Proc. Natl. Acad. Sci. USA.

[78] Liu X., Xing M., Kong C., Fang Z., Yang L., Zhang Y. (2019). Genetic Diversity, Virulence, Race Profiling, and Comparative Genomic Analysis of the *Fusarium oxysporum* f. sp. conglutinans Strains Infecting Cabbages in China. Front. Microbiol..

[79] Glass N.L., Donaldson G.C. (1995). Development of Primer Sets Designed for Use with the PCR to Amplify Conserved Genes from filamentous ascomycetes. Appl. Environ. Microbiol..

[80] White T.J., Bruns T., Lee S., Taylor J. (1990). Amplification and Direct Sequencing of Fungal Ribosomal RNA Genes for Phylogenetics. PCR Protocols.

[81] Gardes M., Bruns T.D. (1993). ITS Primers with Enhanced Specificity for Basidiomycetes—Application to the Identification of Mycorrhizae and Rusts. Mol. Ecol..

[82] Bertrand S., Azzollini A., Schumpp O., Bohni N., Schrenzel J., Monod M. (2014). Multi-Well Fungal Co-Culture for de Novo Metabolite-Induction in Time-Series Studies Based on Untargeted Metabolomics. Mol. BioSyst..

[83] Capodanno E., Moreau S., Levi M., Shimadzu Corporation (2013). Rapid Simultaneous Assay of 23 Mycotoxines in a Variety of Food Samples by UHPLC-MS/MS Using Fast Polarity Switching.

[84] Moreau S., Levi M., Shimadzu Corporation (2014). Highly Sensitive and Rapid Simultaneous Method for 45 Mycotoxins in Baby Food Samples by HPLC-MS/MS Using Fast Polarity Switching.

[85] Abràmoff M.D., Magalhães P.J., Ram S.J. (2004). Image Processing with ImageJ. Biophotonics Int..

[86] Hamzah T.N.T., Lee S.Y., Hidayat A., Terhem R., Faridah-Hanum I., Mohamed R. (2018). Diversity and Characterization of Endophytic Fungi Isolated from the Tropical Mangrove Species, Rhizophora Mucronata, and Identification of Potential Antagonists against the Soil-Borne Fungus, *Fusarium solani*. Front. Microbiol..

