# Supplementary material for: Modulation of Growth and Mycotoxigenic Potential of Pineapple Fruitlet Core Rot Pathogens during In Vitro Interactions
Source: Toxins (Basel). 2024 Aug 7;16(8):344. doi: 10.3390/toxins16080344 (PMC11360085; doi:10.3390/toxins16080344)
Supplement: Supplementary file 1 [file toxins-16-00344-s001.zip › toxins-3123083-supplementary.pdf]

Supplementary Materials: Modulation of Growth and Mycotoxigenic Potential of Pineapple Fruitlet Core Rot Pathogens during In Vitro Interactions

Manon Vignassa, Christian Soria, Noël Durand, Charlie Poss , Jean-Christophe Meile, Marc Chillet and Sabine Schorr-Galindo

Table S1. Identification of fungal species isolated from naturally infected pineapple fruitlets and selected for co-culture bioassays.

Isolate ID	Identification	Fragment size (bp)	Identity (%)	E-value	Locus	GenBank Accession Number
BP429	*Fusarium proliferatum*	610	98.85	0	TEF1-α	MT095058.1
		515	100	0	ITS	MK611678.1
		273	100	0	β-tubulin	MK347275.1
BP369	*Fusarium oxysporum*	304	100	2.0×10^{-157}	TEF1-α	MN784797.1
		472	99.58	0	ITS	MZ722998.1
		235	99.15	3.0×10^{-115}	β-tubulin	MT011050.1
BP462	*Talaromyces stollii*	300	99.67	6.0×10^{-153}	ITS	KP050576.1
		269	99.63	3.0×10^{-136}	β-tubulin	JX315634.1

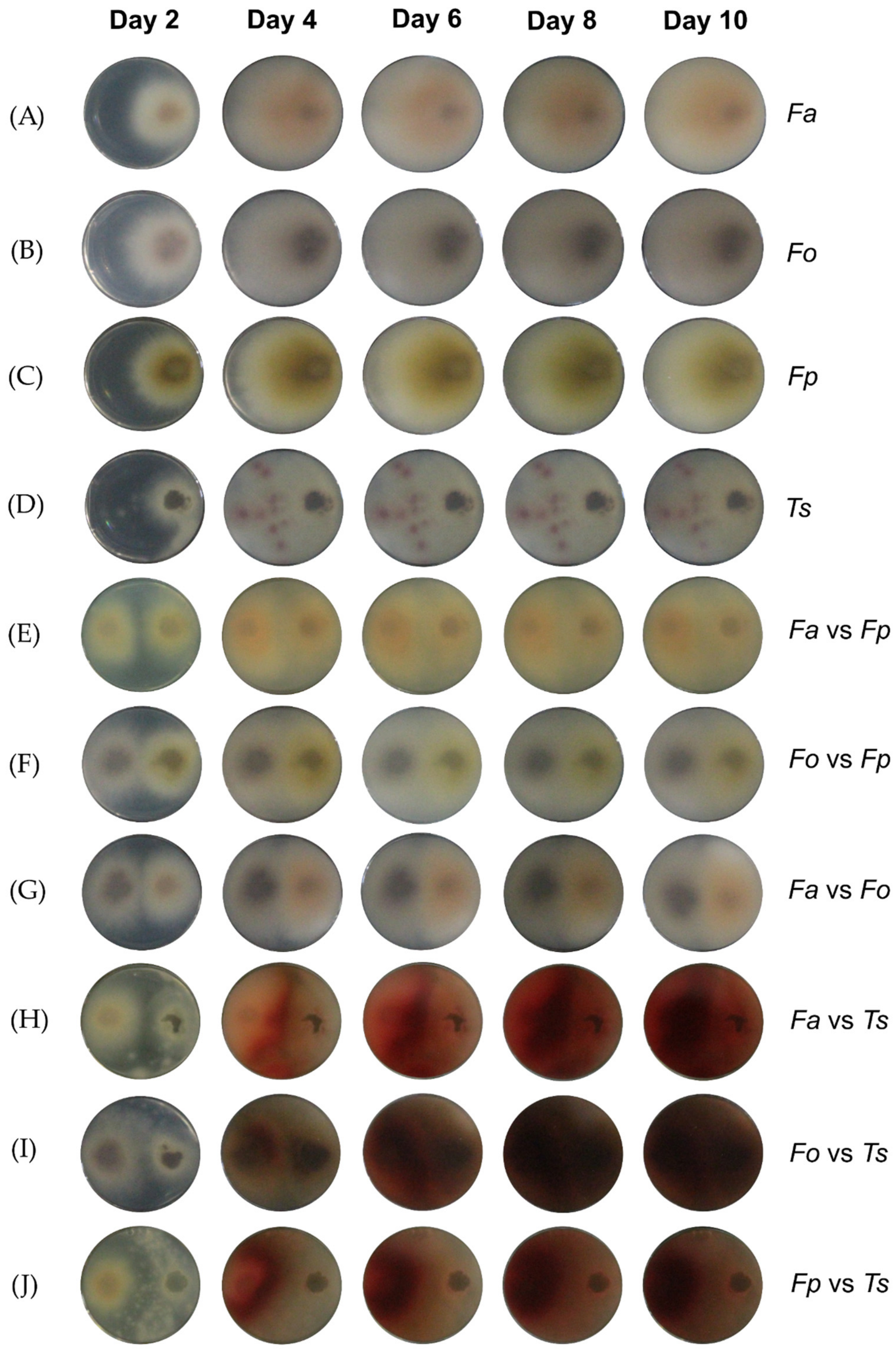

Figure S1. FCR pathogens colony aspects on PDA view from bottom of culture plates after 10 days of incubation in single culture of (A) *Fusarium ananatum* (*Fa*), (B) *Fusarium oxysporum* (*Fo*), (C) *Fusarium proliferatum* (*Fp*) and (D) *Talaromyces stollii* (*Ts*) or in condition of co-culture corresponding to (E) *F. ananatum* versus *F. proliferatum* (*Fa* vs *Fp*), (F) *F. oxysporum* versus *F. proliferatum* (*Fo* vs *Fp*), (G) *F. ananatum* versus *F. oxysporum* (*Fa* vs *Fo*), (H) *F. ananatum* versus *T. stollii* (*Fa* vs *Ts*), (I) *F. oxysporum* versus *T. stollii* (*Fo* vs *Ts*), and (J) *F. proliferatum* versus *T. stollii* (*Fp* vs *Ts*), well diameter = 2 cm.

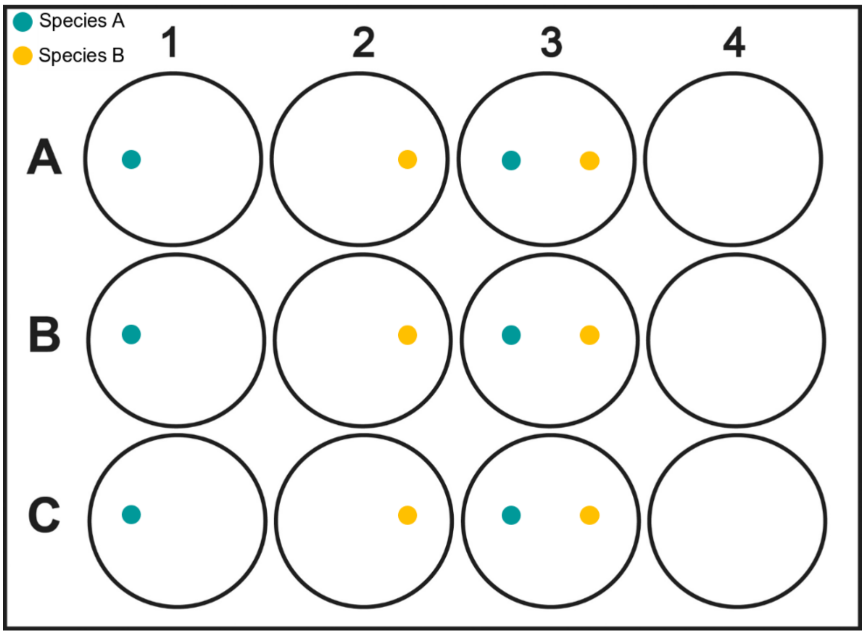

Figure S2. Co-culture procedure performed with fungal species defined as FCR pathogens of pineapple on 12-well culture plates. 1,2: single cultures, 3: co-cultures, 4: non-inoculated wells.
